# Effects of Nrf1 in Hypothalamic Paraventricular Nucleus on Regulating the Blood Pressure During Hypertension

**DOI:** 10.3389/fnins.2021.805070

**Published:** 2021-12-06

**Authors:** Xiao-Jing Yu, Tong Xiao, Xiao-Jing Liu, Ying Li, Jie Qi, Nianping Zhang, Li-Yan Fu, Kai-Li Liu, Yanjun Li, Yu-Ming Kang

**Affiliations:** ^1^Key Laboratory of Environment and Genes Related to Diseases of Education Ministry of China, Department of Physiology and Pathophysiology, Shaanxi Engineering and Research Center of Vaccine, Xi’an Jiaotong University School of Basic Medical Sciences, Xi’an, China; ^2^Department of Cardiology, The Second Clinical Medical College, Shanxi Medical University, Taiyuan, China; ^3^Department of Clinical Medicine, Shanxi Datong University School of Medicine, Datong, China; ^4^Department of Microbiology and Immunology, Shanxi Datong University School of Medicine, Datong, China

**Keywords:** Nrf1, NMDAR, neurotransmitters, hypothalamic paraventricular nucleus, hypertension

## Abstract

The incidence rate and mortality of hypertension increase every year. Hypothalamic paraventricular nucleus (PVN) plays a critical role on the pathophysiology of hypertension. It has been demonstrated that the imbalance of neurotransmitters including norepinephrine (NE), glutamate (Glu) and γ-aminobutyric acid (GABA) are closely related to sympathetic overactivity and pathogenesis of hypertension. N-methyl-D-aspartate receptor (NMDAR), consisting of GluN1 and GluN2 subunits, is considered to be a glutamate-gated ion channel, which binds to Glu, and activates neuronal activity. Studies have found that the synthesis of respiratory chain enzyme complex was affected and mitochondrial function was impaired in spontaneously hypertensive rats (SHR), further indicating that mitochondria is associated with hypertension. Nuclear respiratory factor 1 (Nrf1) is a transcription factor that modulates mitochondrial respiratory chain and is related to GluN1, GluN2A, and GluN2B promoters. However, the brain mechanisms underlying PVN Nrf1 modulating sympathoexcitation and blood pressure during the development of hypertension remains unclear. In this study, an adeno-associated virus (AAV) vector carrying the shRNA targeting rat Nrf1 gene (shNrf1) was injected into bilateral PVN of male rats underwent two kidneys and one clip to explore the role of Nrf1 in mediating the development of hypertension and sympathoexcitation. Administration of shNrf1 knocked down the expression of Nrf1 and reduced the expression of excitatory neurotransmitters, increased the expression of inhibitory neurotransmitters, and reduced the production of reactive oxygen species (ROS), and attenuated sympathoexcitation and hypertension. The results indicate that knocking down Nrf1 suppresses sympathoexcitation in hypertension by reducing PVN transcription of NMDAR subunits (GluN1, GluN2A, and GluN2B), rebalancing PVN excitatory and inhibitory neurotransmitters, inhibiting PVN neuronal activity and oxidative stress, and attenuating sympathetic activity.

## Introduction

The morbidity and mortality caused by hypertension are increasing in past decades. Notably, the overall average age of the first diagnosis of hypertension is decreasing, which means that many young and middle-aged people are suffering from hypertension. Because the pathogenesis of hypertension is quite complicated, and the understanding of its related mechanism is very limited, hypertension can only be controlled by long-term medication. Patients with hypertension also have complications such as kidney and eye diseases. Thus, the prevention and treatment of hypertension are of much importance.

The occurrence and development of hypertension are affected by many factors such as smoking, drinking, genetic factors, excessive sugar intake, high-salt diet, age and other factors. Previous studies on hypertension have mainly focused on the periphery, in which inflammatory response, renin-angiotensin-aldosterone system, non-coding RNA influence, macrophage polarization, etc may cause hypertension. With the deepening of research, recent studies have demonstrated that the paraventricular nucleus of hypothalamus (PVN) plays a critical role in the development of hypertension. Renin-angiotensin system (RAS) is actively involved in the modulation of blood pressure and water-electrolyte homeostasis (Li et al., 2017). Angiotensin II is a key component of RAS, which can be transformed into endothelial cells by angiotensin I. Angiotensin II can also enter the central nervous system to activate sympathetic nerve activity, leading to increased blood pressure (Li et al., 2017). These angiotensin-like sympathetic nerve excitatory pathways are conducted from neurons from the circumventricular organs of the forebrain to the PVN. These pathways further project from the PVN to the rostral ventrolateral medulla or directly extend to the middle and outer cell rows of the spinal cord (Souza et al., 2019). In the central regulation of blood pressure, PVN acts as the key network pivot of the angiotensin neural circuit (Souza et al., 2019). PVN is a unique area located in the forebrain ventricle, which regulates cardiovascular, neuroendocrine and other physiological activities related to homeostasis (Zhou et al., 2019). Norepinephrine (NE) is a potent agonist of α-adrenergic receptors, mainly involved in cardiovascular activities, pain and other physiological functions, and reflects the state of sympathetic nerve excitement. Studies have found that the endogenous NE content in SHR is of high level (Grundt et al., 2009), suggesting that NE in PVN is closely related to the pathogenesis of hypertension.

Sympathetic overactivity is always accompanied with the development of hypertension, and its regulation requires the mediation of neurotransmitters. Currently known key neurotransmitters involved in the regulation of sympathetic nerves are glutamate (Glu) and γ-aminobutyric acid (GABA) (Holbein et al., 2018). Administration of Glu blockers in the brain of spontaneously hypertensive rats (SHR) lowered blood pressure, while micro-administration of Glu in the brain of hypertensive rats induced by two kidneys and one clip (2K1C) method increased blood pressure rapidly (Qiao et al., 2017). GABA can be catalyzed by Glu decarboxylase in nerve endings and has an inhibitory effect. Studies have found that administration of GABA receptor agonists in SHR lowered blood pressure; administration of Glu decarboxylase inhibitors and GABA receptor antagonists increased blood pressure and adrenaline secretion (Dampney et al., 2018). As a Glu-gated ion channel, excessive activation of N-methyl-D-aspartate receptor (NMDAR) has been found to locate outside the synaptic areas and is involved in the up-regulation of neuronal activity and, importantly, increase of blood pressure; while PVN neuronal NMDAR activation also increase blood pressure (Paoletti and Neyton, 2007). The activation of NMDAR is closely related to Glu and GABA function. NMDAR consists two GluN1 subunits and two other subunits varying from GluN2A-D or GluN3A-B. Nrf1 is associated with the transcriptional regulation of GluN1 and GluN2B (Dhar and Wong-Riley, 2009), which are found in most of the NMDARs in neural systems.

Normally, ROS can be maintained in a fixed range, and it increases after receiving stimulation. Excessive generation of ROS causes oxidative stress, damaged nucleic acid structure and protein function, cell damage and even death (Oparka et al., 2016). Studies have found that central oxidative stress is closely related to peripheral sympathetic overactivity (Zhang et al., 2019). Superoxide dismutase (SOD) converts superoxide anion to hydrogen peroxide, which is then converted into water under the catalysis of peroxidase and other antioxidant enzymes (Danková et al., 2019). Nrf1 has been reported to modulate the transcription of several genes related to antioxidant expression such as SOD1 and cytochrome c oxidase subunits (Venugopal and Jaiswal, 1996; Ohtsuji et al., 2008; Dhar et al., 2009; Zhao et al., 2011), and thus may be involved in ROS regulation during the pathophysiology of hypertension. In addition, research found that regions with high oxidative stress have higher levels of glutamatergic and NMDAR-mediated synapses, and that the level of ROS and NMDAR subunits reduces simultaneously when excitatory transmission is suppressed (Wong-Riley M. et al., 1998; Wong-Riley M. T. et al., 1998). During hypertension, both level of ROS and expression of NMDAR increase (Li et al., 2021). It is likely that Nrf1 regulates ROS and NMDAR at the same time.

The pathophysiological process of hypertension may be related to inflammation, neurotransmitter, ROS, etc. The mechanism may be related to the expression of mitochondria and NMDAR subunits. In this experiment, shNrf1 AAV vector was given to bilateral PVN of male rats underwent two kidneys and one clip to explore whether knocking down Nrf1 affects ROS content, neurotransmitter expression, and NMDAR subunits, thereby affecting the development of hypertension.

## Materials and Methods

### Animals

Male Sprague-Dawley (SD) (250–270 g) rats were housed individually in environment with controlled temperature and light: dark cycle. All rats were allowed access to standard chow and tap water *ad libitum*. All rat procedures were approved by the Animal Ethics Committee of Xi’an Jiaotong University and conducted in accordance with the National Institutes of Health Guide for the Care and Use of Laboratory Animals.

### Groups

2K1C procedure was used to establish hypertensive rat model. Rats were divided into four groups following a randomized manner: (1) SHAM + scr (scrambled shRNA was microinjected to bilateral PVN of SHAM rats); (2) SHAM + shNrf1 (the AAV vector carrying Nrf1 shRNA was microinjected to bilateral PVN of SHAM rats); (3) 2K1C + scr (scrambled shRNA was microinjected to bilateral PVN of 2K1C rats); (4) 2K1C + shNrf1 (the AAV vector carrying Nrf1 shRNA was microinjected to bilateral PVN of 2K1C rats). Some of 2K1C and SHAM rats received AAV with scrambled shRNA, and others received AAV with Nrf1 shRNA.

### Hypertensive Rat Model

In this experiment, two kidneys and one clip (2K1C) method was used to construct a hypertensive rat model as mentioned before (Yu et al., 2021). Before the experiment, SD male rats were adaptively fed for 1 week, and blood pressure was tested. Repeat 3–4 times and take the average value. Adapt it to the measurement environment. Before the operation, the rats were fasted and watered continuously (12h), and were anesthetized with 1% sodium pentobarbital in the abdominal cavity. The anesthetized rat was placed on its right side on the bench, and a 2 cm longitudinal incision was made starting at the level of the third lumbar vertebra and 1 cm left to the spine to expose the left kidney. Separate the adipose tissue around the kidney and expose the renal artery. Then an acupuncture needle was placed parallel to the renal artery and ligated with the surgical thread. At this time, it can be seen that the left kidney changes from blood red to yellow, indicating that the ligation position is correct. Remove the acupuncture needles and cause renal artery stenosis. The rats in the sham operation group were only threaded and not ligated, and the rest were the same.

### Recording of Blood Pressure

The systolic blood pressure (SBP) was detected using tail-cuff method every week as previously described (Yu et al., 2021). Un-anesthetized rats were warmed to an ambient temperature of 32°C by placing rats in a holding device mounted on a thermostatically controlled warming plate. Rats were allowed to habituate to this procedure for 3 days prior to each experiment. SBP values were averaged from seven consecutive cycles per day obtained from each rat. At the end of the experiment, rats were anesthetized and placed dorsally on a heated surgical table. An incision along the blood vessels was made in the thigh near groin, and femoral artery was isolated; polyethylene catheters were placed into the femoral artery and advanced into the abdominal aorta for the measurement of SBP. The catheters, filled with 0.1 ml heparin saline (50 units/ml), were connected to a pressure transducer attached to a digital BP monitor and a polygraph. After waiting for 10 min, SBP data were collected for 20 min and averaged.

### Tissue Samples Collection

Rats were decapitated under anesthesia to gather brain and blood tissue. Trunk blood samples were collected in chilled ethylenediaminetetraacetic acid tubes. Plasma samples were separated and stored at −80°C until assayed. The brain specimens were also reserved at −80°C for later analysis (Kang et al., 2011).

### ELISA

Plasma NE were detected using the ELISA kits (Biosource International Inc., Camarillo, California). According to the manufacturer’s descriptions, the standards or sample diluents were added in the appropriate well of microtiter plate precoated with specific antibodies and incubated. Conjugate was added and incubated at 37°C for 1 h and then washed. The reactions were stopped with stop solution and read at 450 nm for NE measurements using a microtiter plate reader (MK3, Thermo Fisher Scientific, United States).

### Real-Time PCR

Real-time PCR was done for Nrf1, GluN1, GluN2A, and GluN2B mRNA in PVN as has been mentioned before (Sriramula et al., 2013). Total RNA was extracted from the ileum (small intestine) with TRIzol reagent (Invitrogen Corporation, United States) and converted to cDNA according to the manufacturer’s protocols. The mRNA concentration in the samples was measured spectrophotometrically and purity of RNA checked by 260/280 ratio. Real-time PCR was carried out using the Mx3005P Detection System (Agilent Technologies, United States).

### Dihydroethidium Staining

Fluorescent-labeled dihydroethidium (DHE) was used to detect superoxide production in the PVN. Coronal sections (18 mm) were incubated with DHE (0.05 mM) for 30 min at 37^°^C. Sections containing PVN were then rinsed with PBS (0.01 M) three times and were then observed using a Nikon epifluorescence microscope (Bai et al., 2017).

### Western Blotting

The PVN tissue was homogenized in lysis buffer and Western blotting was performed as previously described (Yu et al., 2021). The protein concentration was measured and loaded onto an SDS-PAGE gel and then transferred to a polyvinylidene fluoride membrane. The membrane was then incubated overnight at 4°C with the primary antibodies including: Nrf1 (12482-1-AP, Proteintech), GluN1 (A7677, ABclonal), GluN2A (NB300-105, Novus Biologicals), GluN2B (NB300-106, Novus Biologicals). They were purchased from Santa Cruz Biotechnology. After washing with wash buffer four times for 10 min each time, blots were then incubated for 1 h with secondary antibody (Santa Cruz Biotechnology) labeled with horseradish peroxidase. Protein loading was controlled by probing all blots with β-actin antibody (Thermo Scientific, United States) and normalizing their protein intensities to that of β-actin. Band densities were visualized with Bio-Rad Chemi Doc XRS + and analyzed using ImageJ (NIH).

### Immunofluorescence

Rats were anesthetized and fixed with perfusion using 0.01M phosphate-buffered solution (PBS) into the left ventricle first and then with 4% paraformaldehyde. The brain samples were collected and soaked in 4% paraformaldehyde and then 30% sucrose. Tissue microdissection was used to separate the PVN tissue. Immunofluorescence was performed to observe Fra-like immunoreactivity (Fra-LI, a marker of chronic neuronal activation), tyrosine hydroxylase (TH), GABA, Glu. Antibody origin and proportion: Fra-LI (sc-253, Santa Cruz), TH (sc-25269, Santa Cruz), GABA (ab86186, Abcam), Glu (ab9440, Abcam). Frozen sections containing PVN were permeated with 0.3% Triton-X for 30 min at 37°C prior to incubation with antibody at 4°C overnight. After washing with PBS three times, sections containing PVN were incubated with Alexa 488-labeled secondary antibody (Invitrogen) and Alexa 594-labeled secondary antibody (Invitrogen) for 120 min at 37°C. Briefly, frozen brain sections were incubated with 0.3% Triton-X for 30 min at 37°C, and primary antibody at 4°C overnight. After being washed with PBS, incubated with corresponding secondary antibody for 120 min at 37°C, sections were covered with coverslips and imaged using Nikon microscopy (Kang et al., 2011).

### Statistical Analysis

Data were presented as means ± SEM. Data were analyzed using one-way ANOVA with Tukey’s multiple comparison tests except the SBP data, which were analyzed using repeated-measures ANOVA. *P* < 0.05 were set as significant.

## Results

### Results of Blood Pressure and Plasma Norepinephrine

**FIGURE 1:**
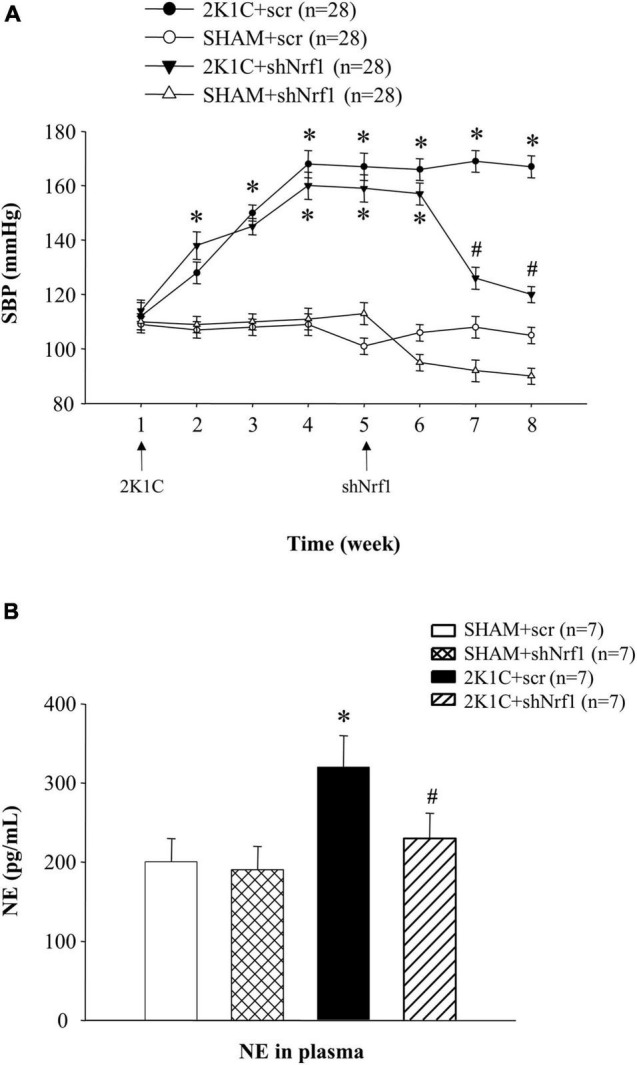
Results of blood pressure and plasma NE in different groups. **(A)** Effects of knocking down Nrf1 on SBP of hypertensive rats. *P < 0.05 vs. control animals (SHAM + scr or SHAM + shNrf1). ^#^*P* < 0.05 2K1C + shNrf1 vs. 2K1C + scr. **(B)** Effects of knocking down Nrf1 on NE in hypertensive rats. *P < 0.05 vs. control animals (SHAM + scr or SHAM + shNrf1). ^#^*P* < 0.05 2K1C + shNrf1 vs. 2K1C + scr.

From Figure 1A, it is demonstrated that SBP in 2K1C groups was higher than that in SHAM group, indicating that the rat model was successfully established. The SBP in sham operation group did not change significantly. After successful modeling of hypertensive rats, shNrf1 AAV vector was administered to the PVN and SBP was monitored. When compared with the 2K1C + scr group, SBP decreased after Nrf1 was knocked down.

Plasma NE can reflect the state of sympathetic nerve excitement. Blood samples were collected after administration, and ELISA was used to detect the plasma NE. In Figure 1B, the plasma NE expression in 2K1C + scr group increased significantly in comparison with SHAM groups, suggesting sympathetic nerve activity was enhanced in hypertensive rats. Compared with the 2K1C + scr group, plasma NE of 2K1C + shNrf1 group was significantly reduced, indicating that knocking down Nrf1 attenuated the sympathetic nerve activity.

### Results of Nrf1 Protein and mRNA Expression in the Paraventricular Nucleus

**FIGURE 2:**
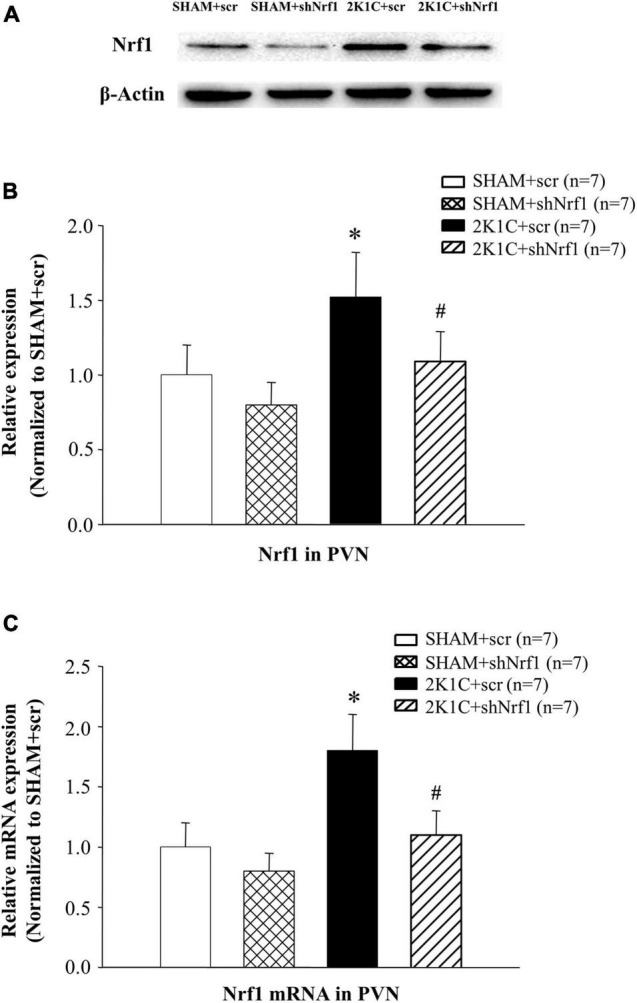
Translation and transcription level of Nrf1 in PVN of hypertensive rats. **(A)** Expression of Nrf1 protein in PVN in different groups detected by Western blotting; **(B)** Group data for Nrf1 protein expression in PVN; **(C)** Group data for the mRNA expression of Nrf1 in PVN. *P < 0.05 vs. control animals (SHAM + scr or SHAM + shNrf1). ^#^*P* < 0.05 2K1C + shNrf1 vs. 2K1C + scr.

It can be seen from Figure 2 that Nrf1 protein expression and mRNA levels were significantly reduced after the shNrf1 was administered to the PVN, indicating that the administration of shNrf1 was successful. Nrf1 in 2K1C + scr group increased in comparison with SHAM groups.

### Effects of Nrf1 on Reactive Oxygen Species in the Paraventricular Nucleus of Hypertensive Animals

**FIGURE 3:**
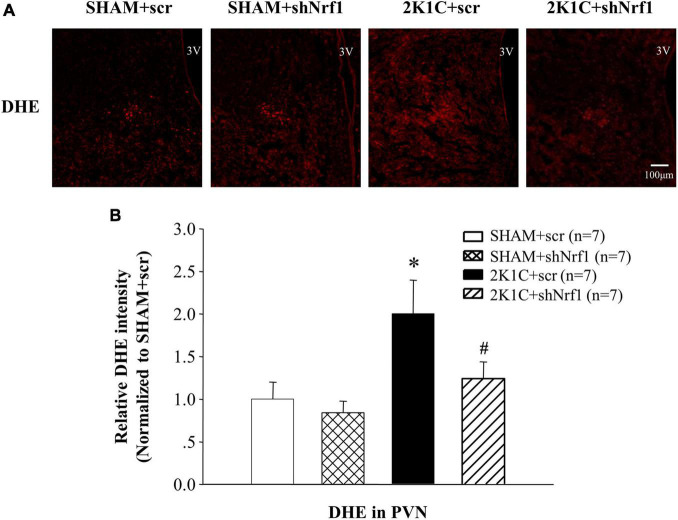
Effects of knocking down of Nrf1 on ROS in PVN of hypertensive rats. **(A)** DHE staining displaying superoxide anion production in the PVN; **(B)** Fluorescent intensity of DHE in PVN. *P < 0.05 vs. control animals (SHAM + scr or SHAM + shNrf1). ^#^*P* < 0.05 2K1C + shNrf1 vs. 2K1C + scr.

DHE staining was used to measured ROS in the PVN. It can be seen from Figure 3 that ROS in the PVN of 2K1C + scr group was significantly increased in comparison with SHAM groups; ROS in the PVN of the 2K1C + shNrf1 group significantly reduced as compared with the 2K1C + scr group.

### Effects of Knockdown of Nrf1 on Neurotransmitters in the Paraventricular Nucleus of Hypertensive Rats

**FIGURE 4:**
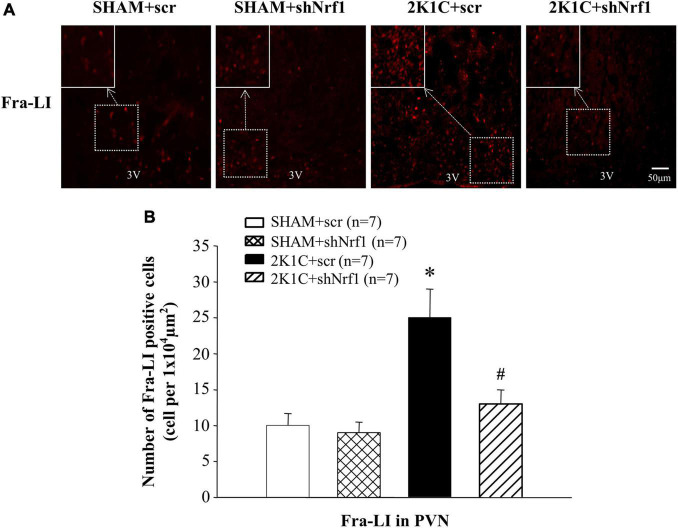
Effects of knocking down of Nrf1 on Fra-LI expression in PVN of hypertensive rats. **(A)** Immunofluorescence for Fra-LI in PVN in different groups. **(B)** Changes of number of Fra-LI positive cells in PVN. *P < 0.05 vs. control animals (SHAM + scr or SHAM + shNrf1). ^#^*P* < 0.05 2K1C + shNrf1 vs. 2K1C + scr.

Sympathetic overactivity is closely related to the development of hypertension, and its regulation requires the mediation of neurotransmitters. Immunofluorescence was used to measure Fra-LI, TH, Glu, and GABA in PVN of rats. Fra-LI in PVN of the 2K1C + scr group increased significantly in comparison with SHAM groups, indicating that hypertension may be accompanied by PVN neuronal excitation. Fra-LI in PVN of the 2K1C + shNrf1 group was significantly reduced in comparison with 2K1C + scr group (Figure 4).

**FIGURE 5:**
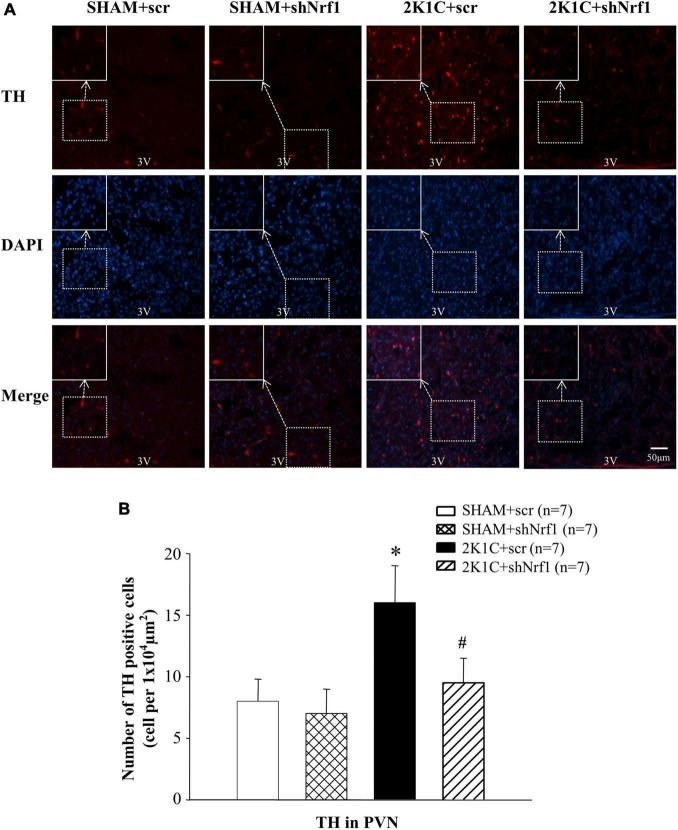
Effects of knocking down of Nrf1 on TH expression in PVN of hypertensive rats. **(A)** Immunofluorescence for the expression of TH in PVN in different groups. **(B)** Changes of number of TH positive cells in PVN. *P < 0.05 vs. control animals (SHAM + scr or SHAM + shNrf1). ^#^*P* < 0.05 2K1C + shNrf1 vs. 2K1C + scr.

TH is widely expressed in neurons and is the first step to mediate the synthesis of catecholamines (dopamine, NE, and epinephrine). Compared with the SHAM groups, TH expression in PVN of 2K1C + scr group increased significantly, indicating that the expression of TH in the PVN is affected in the hypertension. Compared with the 2K1C + scr group, the TH expression in the PVN of the 2K1C + shNrf1 group was significantly reduced (Figure 5). It shows that knocking down Nrf1 reduced TH, thereby affecting the synthesis of NE, weakening sympathetic nerve activity, and lowering blood pressure.

**FIGURE 6:**
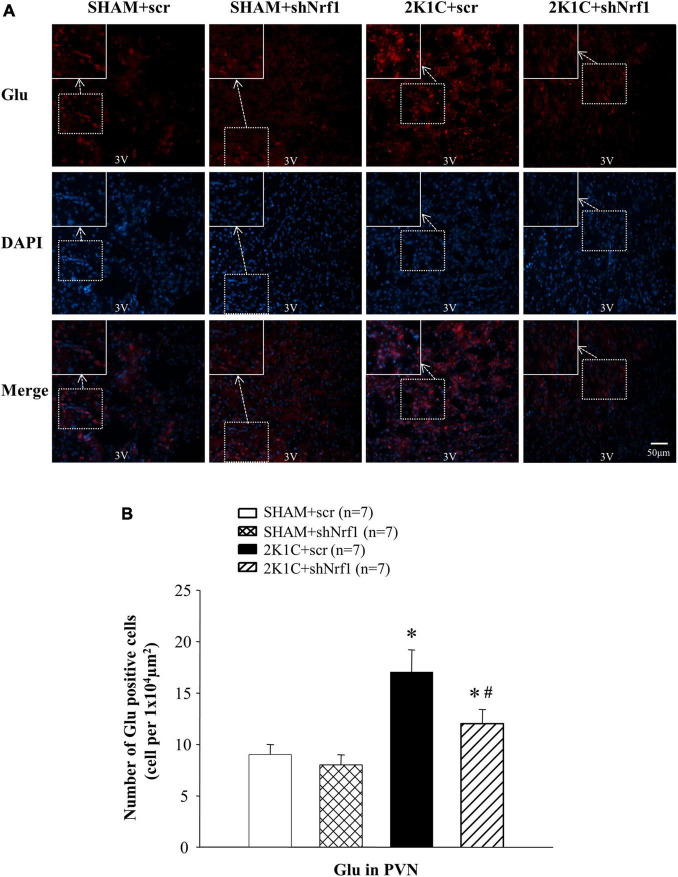
Effects of knocking down of Nrf1 on Glu in PVN of hypertensive rats. **(A)** Immunofluorescence for the expression of Glu in PVN in different groups. **(B)** Changes of the number of Glu positive cells in PVN. *P < 0.05 vs. control animals (SHAM + scr or SHAM + shNrf1). ^#^*P* < 0.05 2K1C + shNrf1 vs. 2K1C + scr.

**FIGURE 7:**
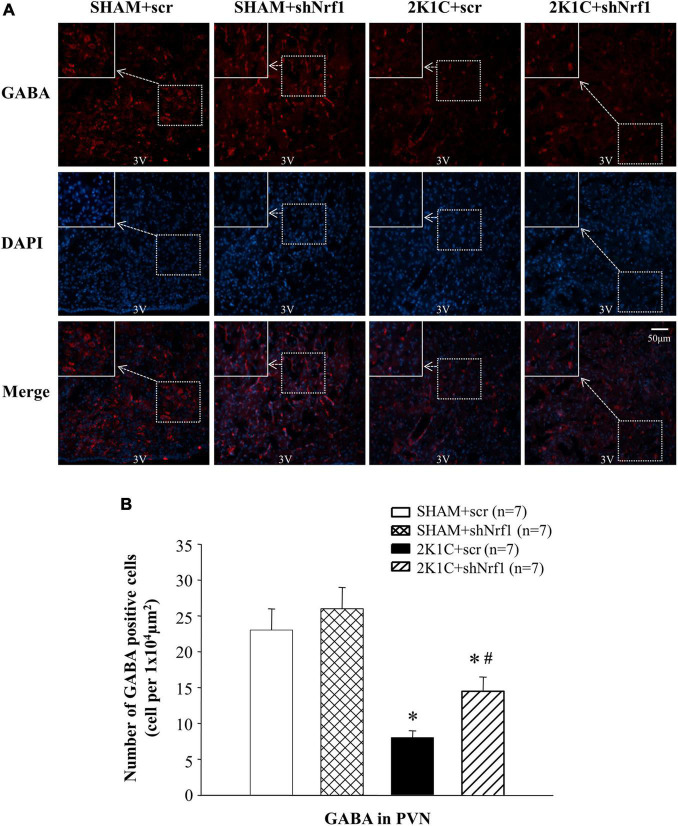
Effects of knocking down of Nrf1 on GABA expression in PVN of hypertensive rats. **(A)** Immunofluorescence for the expression of GABA in PVN in different groups. **(B)** Changes of the number of GABA positive cells in PVN. *P < 0.05 vs. control animals (SHAM + scr or SHAM + shNrf1). ^#^*P* < 0.05 2K1C + shNrf1 vs. 2K1C + scr.

Glu is an excitatory neurotransmitter, which can bind to ionotropic receptors to increase the excitability of neurons. GABA is an inhibitory neurotransmitter that can lower blood pressure. The number of Glu positive cells in the PVN of 2K1C groups was significantly increased in comparison with SHAM groups (Figure 6), and the number of GABA positive cells of 2K1C groups was reduced in comparison with SHAM groups; when compared with the 2K1C + scr group, the expression of Glu in PVN of 2K1C + shNrf1 animals was significantly decreased (Figure 6), and the expression of GABA in PVN of 2K1C + shNrf1 animals was increased (Figure 7).

### Effects of Knocking Down Nrf1 on the Protein and mRNA Expression of N-Methyl-D-Aspartate Receptor Subunits in the Paraventricular Nucleus of Hypertensive Rats

**FIGURE 8:**
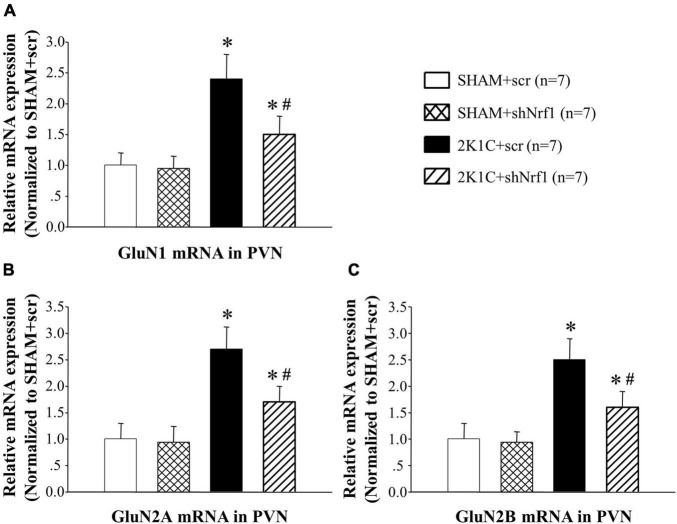
Effects of knocking down of Nrf1 on GluN1, GluN2A, and GluN2B mRNA expression in PVN of hypertensive rats by real-time PCR. **(A)** Group data for GluN1 mRNA expression in PVN; **(B)** group data for GluN2A mRNA in PVN; **(C)** group data for GluN2B mRNA in PVN. *P < 0.05 vs. control animals (SHAM + scr or SHAM + shNrf1). ^#^*P* < 0.05 2K1C + shNrf1 vs. 2K1C + scr.

**FIGURE 9:**
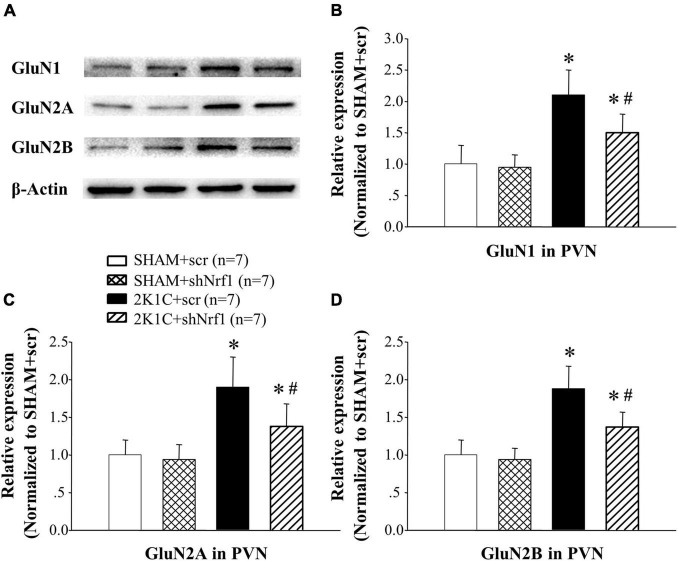
Effects of knocking down of Nrf1 on the protein expressions of GluN1, GluN2A, and GluN2B in PVN of hypertensive rats. **(A)** Western blotting for the protein expressions of GluN1, GluN2A, and GluN2B in PVN in different groups; **(B)** group data for GluN1 protein expression in PVN; **(C)** group data for GluN2A protein expression in PVN; **(D)** group data for GluN2B protein expression in PVN. *P < 0.05 vs. control animals (SHAM + scr or SHAM + shNrf1). ^#^*P* < 0.05 2K1C + shNrf1 vs. 2K1C + scr.

In Figures 8, 9, GluN1, GluN2A, and GluN2B protein expression and mRNA in PVN of 2K1C groups increased significantly in comparison with SHAM groups; GluN1, GluN2A, and GluN2B protein expression and mRNA decreased in PVN of 2K1C + shNrf1 group in comparison with 2K1C + scr group. This shows that knocking down Nrf1 may affect GluN1, GluN2A, and GluN2B of NMDAR subunits to lower blood pressure.

## Discussion

The PVN is one of the important central structures that regulate sympathetic outflow and blood pressure. In the central regulation of blood pressure, the PVN acts as the key network center of the angiotensin neural circuit (Souza et al., 2019). Studies have found that some sympathetic nerve excitatory pathways can project from PVN to the rostral ventrolateral medulla of the brainstem or directly extend to the intermediolateral cell column of the spinal cord (Souza et al., 2019). Sympathetic nerve excitement is very closely associated to hypertension, and the PVN plays a certain role in regulating sympathetic nerve overactivity. Therefore, it is indicated that the development of hypertension requires the participation of PVN. As an important neurotransmitter sympathetic nerve ending, NE participates in sympathetic nerve regulation to constrict blood vessels and increase blood pressure (Janssen et al., 1993; Turcani, 2008). In this study, ELISA was used to detect the expression of plasma NE. Compared with sham controls, the NE of hypertensive rats was found to be significantly higher. After shNrf1 was administered to the bilateral PVN, the NE was decreased.

This study used 2K1C to construct a hypertensive rat model. The hypertension model constructed by this method is stable and has a high successful rate. The rat blood pressure was detected every week after the surgery. The blood pressure increased after the hypertensive model was established. ShNrf1 encapsulated with AAV was administered into the PVN, and the blood pressure was detected after the operation. The results of the experiment demonstrated that the blood pressure began to decrease after the administration, indicating that PVN administration of shNrf1 bilaterally decreased blood pressure of the 2K1C rats. After that, using Western blotting and real-time PCR, we found that the PVN Nrf1 of hypertensive rats decreased after shNrf1 administration, indicating that the experimental administration was successful. Compared with sham controls, the expression of Nrf1 in the 2K1C group was found to be increased, and this might be mediated *via* the oxidative stress response in the PVN during hypertension, that is, the responsiveness of Nrf1 increases during hypertension.

TH is the rate-limiting enzyme for bio-synthesis of NE (Nagatsu and Nagatsu, 2016). It is widely expressed in neurons in the brain and is the first step to mediate the synthesis of catecholamines (dopamine, NE, and epinephrine) (Daubner et al., 2011). This study used immunofluorescence to detect TH protein in the hypothalamus of the rats. The results of the study found that the expression of TH in the brain of the hypertensive rats was increased, compared with sham groups. After shNrf1 was given, the expression of TH was significantly decreased. It shows that shNrf1 can reduce the expression of TH in the brain and then affect the synthesis of NE to lower blood pressure.

TH can mediate the synthesis of catecholamines, and catecholamines regulate Glu and GABA. Glu and GABA are both amino acid neurotransmitters and participate in sympathetic nerve regulation. Glu can bind to ionotropic receptors to increase neuronal excitability (Qiao et al., 2017). Studies have found that micro-administration of Glu into the brain of hypertensive rats by 2K1C method can rapidly increase blood pressure (Qiao et al., 2017); GABA is an important inhibitory neurotransmitter in the PVN, and administration of GABA receptor agonists can lower blood pressure (Dampney et al., 2018). This study used immunofluorescence method to measure the expression of GABA and Glu in PVN. We showed that compared with the sham rats, the expression of GABA in the brain of rats in the 2K1C group was reduced, and the expression of Glu was increased. After shNrf1 was administered, the expression of GABA in the brain of the rats increased, and the expression of Glu was reduced. Our study demonstrated that knocking down Nrf1 in PVN may reduce the expression of Glu and increase the expression of GABA to rebalance of inhibitory and excitatory neurotransmitters in hypertensive rats, thereby reducing blood pressure. But Nrf1 may not directly regulate the expression of Glu and GABA. Rather, it could be an indirect effect as the consequence of down-regulation of NMDAR after Nrf1 knockdown in the PVN.

NMDAR is widely distributed in neurons and is also the main binding site for Glu (Qiao et al., 2017). Studies have found that Glu can cause neurotoxicity by activating NMDA receptors, which in turn leads to neuronal damage (Paoletti and Neyton, 2007). NMDAR is also related to encephalitis and myocardial infarction (Danysz and Parsons, 2003; Olivares et al., 2012; Wang et al., 2015; Jannesar et al., 2020). Our recent study demonstrated that the expression level of NMDAR subunits is elevated during hypertension, and chronic blockade of NMDAR subunit 2A (GluN2A) in the PVN alleviates hypertension (Li et al., 2021). Studies have found that Nrf1 is functionally related to the promoters of GluN1 and GluN2B. We thus tested if knocking down Nrf1 can modulate the increased expression level of NMDAR subunits. In this experiment, Western blotting and real-time PCR were used to detect the expression of GluN1, GluN2A and GluN2B in the PVN. The results of this study showed that the expressions of GluN1, GluN2A, and GluN2B in PVN of hypertensive animals was increased. After shNrf1 was given, the expressions of GluN1, GluN2A, and GluN2B in PVN were significantly reduced, consistent with the results of the literature.

Increased ROS production can be found in the PVN of hypertensive animals (Wang et al., 2018, 2021; Yang et al., 2020). The oxidative stress caused by the imbalance of ROS production and removal contributes to the advancement of inflammatory responses and hyperactivity of sympathetic nerves, and promotes the progression of hypertension. Nrf1 can act on the subunits of the respiratory chain enzyme complex, which in turn affects mitochondria and modulate the ROS production (Zhang and Xiang, 2016). In our study, we measured the ROS level by DHE staining, and found high ROS level in the PVN of hypertensive rats while it decreased after Nrf1 knock-down. It is consistent with the literature which suggest that Nrf1 is involved in the transcription of SOD1 and cytochrome c oxidase subunits (Venugopal and Jaiswal, 1996; Ohtsuji et al., 2008; Dhar et al., 2009; Zhao et al., 2011), which are genes related to antioxidant expression. Our result suggested that knocking down Nrf1 can reduce the ROS level in the PVN of hypertensive rats, which contributes to the alleviation of blood pressure.

## Conclusion

The results of this study indicate that knocking down Nrf1 improves sympathoexcitation and hypertension by reducing PVN transcription of NMDAR subunits (GluN1, GluN2A, and GluN2B), and rebalancing PVN excitatory and inhibitory neurotransmitters, and inhibiting PVN neuronal activity and oxidative stress, and attenuating sympathetic activity. This study provides insights into the central mechanism underlying the mediating role of Nrf1 in the development of hypertension, implicating that the Nrf1 may represent a potential target for prevention and treatment of hypertension.

## Data Availability Statement

The raw data supporting the conclusions of this article will be made available by the authors, without undue reservation.

## Ethics Statement

The animal study was reviewed and approved by Institutional Animal Ethics Committee of Xi’an Jiaotong University.

## Author Contributions

Y-MK and X-JY designed the study. TX and X-JY performed all experiments. Y-MK, X-JY, and TX performed the data analysis and drafted the manuscript. Y-MK, X-JY, and X-JL participated in data analysis. Y-MK, X-JY, X-JL, YL, JQ, NZ, L-YF, K-LL, and YJL critically revised the manuscript. All authors reviewed the final manuscript.

## Conflict of Interest

The authors declare that the research was conducted in the absence of any commercial or financial relationships that could be construed as a potential conflict of interest.

## Publisher’s Note

All claims expressed in this article are solely those of the authors and do not necessarily represent those of their affiliated organizations, or those of the publisher, the editors and the reviewers. Any product that may be evaluated in this article, or claim that may be made by its manufacturer, is not guaranteed or endorsed by the publisher.

## Abbreviations

PVN: hypothalamic paraventricular nucleus
ROS: reactive oxygen species
Nrf1: nuclear respiratory factor 1
RAS: renin-angiotensin system
Glu: glutamate
GABA: γ -aminobutyric acid
NE: Norepinephrine
SOD: Superoxide dismutase
NMDAR: N-methyl -D-aspartate receptor
SD: Sprague-Dawley
2K1C: two kidneys and one clip
SBP: systolic blood pressure
DHE: dihydroethidium
TH: tyrosine hydroxylase
AAV: adeno-associated virus
SHR: spontaneously hypertensive rats
Fra-LI: Fra-like immunoreactivity.
